# Non-cardiomyocytes in the heart in embryo development, health, and disease, a single-cell perspective

**DOI:** 10.3389/fcell.2022.873264

**Published:** 2022-10-31

**Authors:** Shuangyuan Ding, Xingwu Zhang, Hui Qiu, Jiaoyang Wo, Fengzhi Zhang, Jie Na

**Affiliations:** ^1^ School of Medicine, Tsinghua University, Beijing, China; ^2^ Center for Life Sciences, Tsinghua University and Peking University, Beijing, China; ^3^ School of Life Sciences, Tsinghua University, Beijing, China; ^4^ Central Laboratory, First Hospital of Tsinghua University, Beijing, China

**Keywords:** non-cardiomyocyte, heart development, heart disease, single-cell analysis, cardiac fibroblast, endothelial cell, cardiac macrophage

## Abstract

Recent single-cell atlases of the heart gave unprecedented details about the diversity of cell types and states during heart development in health and disease conditions. Beyond a profiling tool, researchers also use single-cell analyses to dissect the mechanism of diseases in animal models. The new knowledge from these studies revealed that beating cardiomyocytes account for less than 50% of the total heart cell population. In contrast, non-cardiomyocytes (NCMs), such as cardiac fibroblasts, endothelial cells, and immune cells, make up the remaining proportion and have indispensable roles in structural support, homeostasis maintenance, and injury repair of the heart. In this review, we categorize the composition and characteristics of NCMs from the latest single-cell studies of the heart in various contexts and compare the findings from both human samples and mouse models. This information will enrich our understanding of the cellular basis of heart development and diseases and provide insights into the potential therapeutic targets in NCMs to repair the heart.

## Introduction

The heart is the first solid organ to form and function in mammalian embryo development. A deep understanding of the mechanism behind cardiac cell fate decision and heart structure formation will shed light on eliminating congenital heart diseases. The realization of this ambitious vision needs a comprehensively comprehending of spatiotemporal changes in each cardiac cellular composition. On the other side, cardiovascular diseases (CVDs) are the leading cause of death in adults worldwide (Virani et al., 2021). A clear picture of the transcriptomic changes in different cardiac cell types with higher resolution will enlighten the discovery of new therapeutic targets and intervention strategies. Bulk gene-quantification methods (such as bulk RNA sequencing and qPCR), even with cell purification (such as fluorescence-activated sorting), can only give information on limited cell types due to the availability of specific surface markers. Moreover, the bulk analyses masked the heterogeneity within a cell population and may miss the rare and unknown cell types. Single-cell RNA sequencing (scRNA-seq) overcomes these restrictions by providing the transcriptome of every single cell in a given specimen (Picelli et al., 2014; Zheng et al., 2017).

The human linear heart tubes start beating about 22 days after fertilization, followed by rapid growth and rightward looping. This critical cardiac morphological transformation relies on recruiting NCMs (Mjaatvedt et al., 2001; Zaffran et al., 2004; Waldo et al., 2005; Kelly, Buckingham, and Moorman 2014). In humans, the four-chambered fetal hearts form at 4.5 post-conception weeks (PCWs), with thickened ventricle walls consisting of endocardium, myocardium, and epicardium layers (Brade et al., 2013). Mouse studies have revealed that progenitor cells of the three layers have different developmental origins at the cardiac crescent stage, and each layer has its unique formulas of multiple cell types and states (Meilhac and Buckingham 2018). The heterogeneous and dynamic nature of the embryonic cardiac cell population during heart development represented an excellent model for single-cell analysis.

**FIGURE 1:**
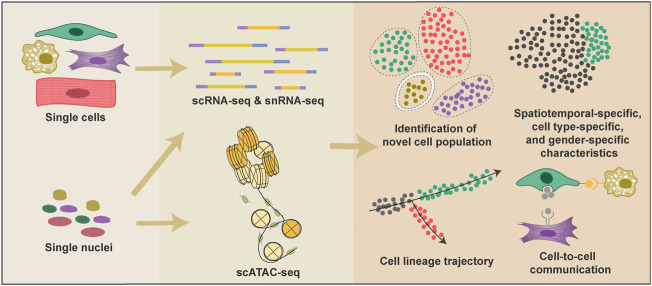
Applications of single-cell technologies and analyses in heart research. Single-cell technologies have many advantages over conventional bulk-based gene quantification methods. Both cells and nuclei can be used to generate single-cell transcriptional information, and nuclei are also adopted in scATAC-seq to provide epigenetic information. Single-cell technologies provide information to classify cell types and proportions under various conditions; reconstruct cell fate change trajectories during heart development and diseases; reveal the spatiotemporal-specific, cell-type specific, and gender-specific characteristics of cardiac cells; detect potential cell-cell interactions.

In the past 5 years, researchers have used both scRNA-seq and single-nucleus RNA sequencing (snRNA-seq) technologies to profile the cellular atlas of human hearts from 4.5 PCWs to adult (Asp et al., 2019; Cui et al., 2019; Litviňuková et al., 2020; Tucker et al., 2020). Single-cell transcriptomic information enables researchers to identify new cell types and different cell states, investigate cell-to-cell communications, and infer cell fate trajectories (Figure 1). Distinct anatomical structures of the heart are highly specialized in their physiological functions; thus, almost every cell type can be divided into multiple subpopulations depending on their anatomical location (Asp et al., 2019). Although single-cell spatial transcriptome is not yet available, the distribution of various cell types in different heart regions can be inferred through the deconvolution algorithm combined with single-molecular fluorescence *in situ* hybridization (Asp et al., 2019). Moreover, researchers also used epigenetic technology such as sing-cell assay for transposase-accessible chromatin sequencing (scATAC-seq) in elucidating the mechanism underlies the mouse heart development and diseases (Jia et al., 2018; Wang Y et al., 2020; Wang et al., 2021) (Figure 1).

Several excellent reviews have summarized the single-cell atlas and the state-of-art technologies to map the heart (Paik et al., 2020; Zhou and Wang 2020a; Author and Wang et al., 2020b). In this review, we focus on the non-cardiomyocyte population of the human heart, including fibroblasts, ECs, and macrophages, under various circumstances, such as in normal heart and congenital and adult heart diseases (Suryawanshi et al., 2020; Wang L. et al., 2020; Hill et al., 2022; Chaffin et al., 2022). Beyond serving as a rich resource, these sing-cell RNA data provide insights into the cell-type-targeted intervention of heart diseases and new potential therapeutic targets and biomarkers for heart diseases.

## The composition of non-cardiomyocytes in the heart

**FIGURE 2:**
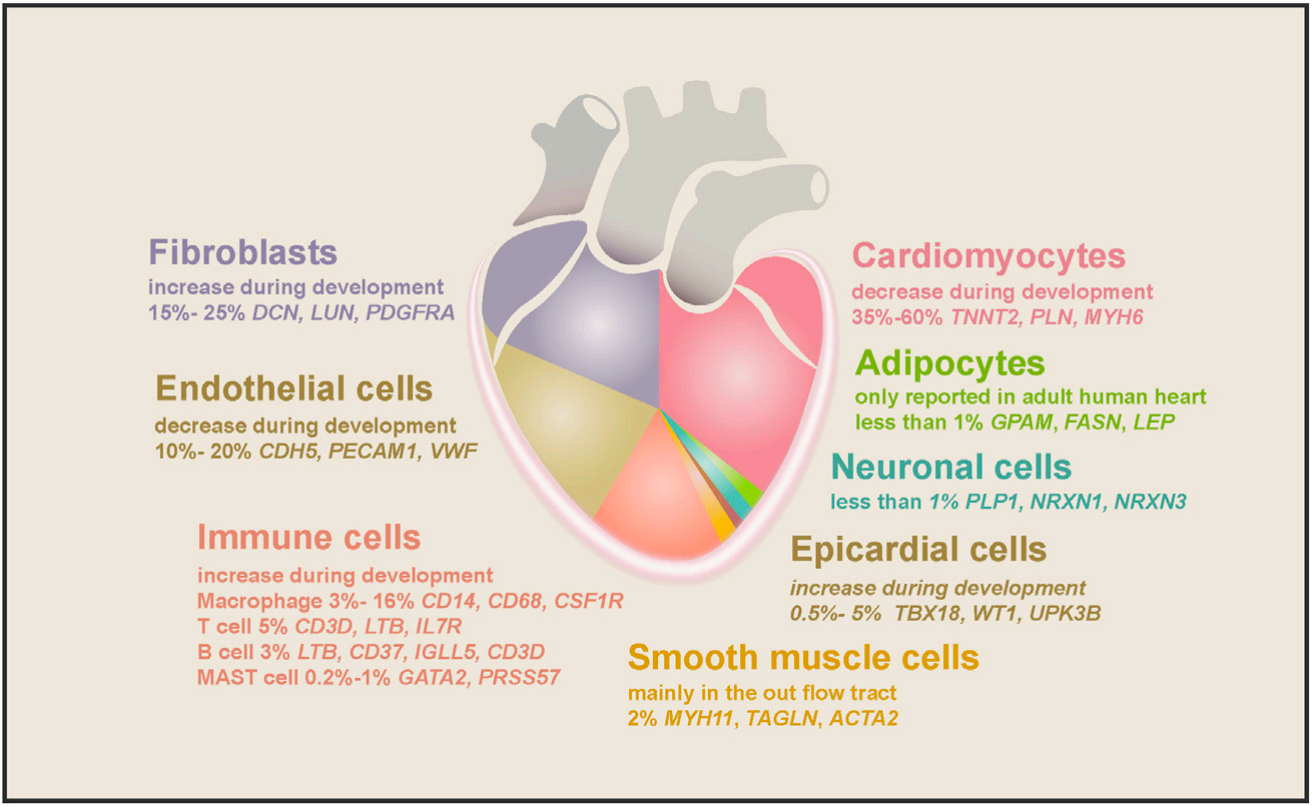
The cell types found in the human heart and their marker genes. Cell types and their proportion range in embryonic and adult hearts are depicted in different colors. As the largest population in the human heart, cardiomyocytes comprise half of the total cells, decreasing during embryonic heart development. The second-largest cell population is fibroblasts, increasing during heart development and aging. ECs accounted for 10%–20% of cardiac cells, and their proportion decreased gradually as the heart matured. Macrophages are the largest cardiac immune cell population, making up half of the total immune cells. SMCs, epicardial cells, and neuronal cells consist of 2%, 0.5%–5%, and less than 1% of cell populations in the heart, while adipocytes were only found in the adult heart.

The single-cell analyses revealed that NCMs account for about half of the total cells in the human heart. However, the exact proportion varies depending on the sequencing technology adopted, the developmental stage of the heart, and the chamber selected. Just as Cui et al. (2019) *.* reported that ∼40% of cells in the 5–7 PCWs embryonic heart are NCMs, while Litviňuková et al. (2020) found ∼70% of cells in the adult atrium and 50% of cells in the ventricle are NCMs. NCMs consist of at least dozens of cell types, such as fibroblast-like cells, endothelial cells (ECs), immune cells (mainly macrophages), and epicardial cells (EPCs) (Figure 2). Each cell type expresses a unique set of marker genes and has been shown to regulate heart morphogenesis, homeostasis, structure, contractility, conductivity, etc. (Asp et al., 2019; Cui et al., 2019; Litviňuková et al., 2020; Tucker et al., 2020).

## Fibroblasts in the heart

**FIGURE 3:**
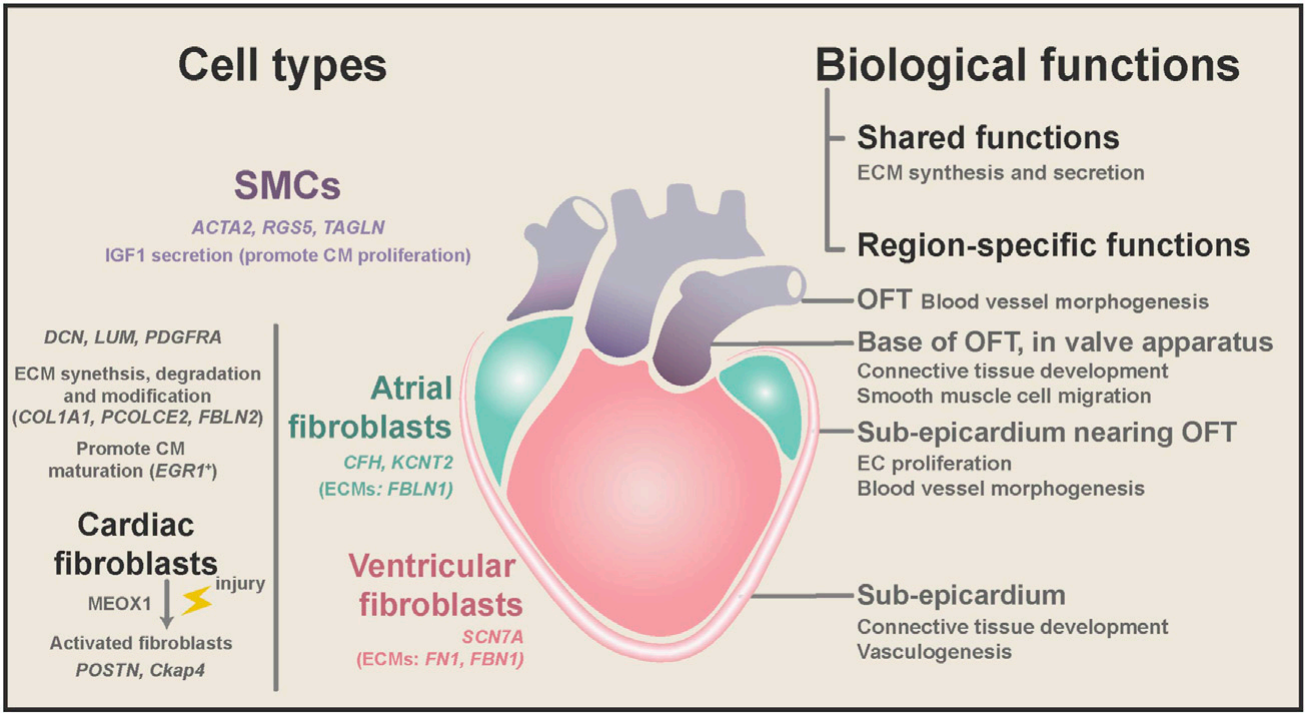
Region-specific characteristics of fibroblasts and SMCs, and their biological functions during homeostasis and diseases. Both cardiac fibroblasts and SMCs have ECM synthesis and secretion ability, while fibroblasts are mainly located in the heart free-wall and SMCs from the OFT. SMCs might promote embryonic CM proliferation by secreting IGF1. Cardiac fibroblasts are involved in the degradation and modification of ECM and may promote CM maturation in the embryonic heart. Cardiac fibroblasts expressed chamber-specific genes and ECM genes. In diseases, cardiac fibroblasts can be activated and cause fibrosis and CM dysfunction. Spatial transcriptome analysis found that cardiac fibroblasts and SMCs have different gene ontology terms depending on their anatomical location.

As the largest NCM population in the human heart (15%–30% in all cell population), fibroblasts (*DCN*, *DLK1*, *PDGFRA,* and *LUM*) play critical roles in heart hemostasis and disease (Zhou and Pu 2016; Cui et al., 2019; Tucker et al., 2020; Wang et al., 2021) (Figure 3). Cardiac fibroblasts are indispensable for heart structure and mechanical support, and their ratio increases from 15% at 7 weeks to 25% in adults (DeLaughter et al., 2016; Cui et al., 2019). Fibroblasts are characterized by their mesenchymal morphology and enriched expression of extracellular matrix (ECM) genes (e.g., *COL1A1*, *ELN*, *OGN*, and *SPARC*) (Tallquist and Molkentin 2017; Asp et al., 2019; Cui et al., 2019; Tucker et al., 2020). Ablation of cardiac fibroblasts in the mouse heart will substantially reduce type I and type III collagen deposition, which is perinatally lethal (Smith et al., 2011; Acharya et al., 2012). Recently, Litviňuková et al. (2020) found that specific fibroblast subtypes also express genes involved in ECM degradation and modification (*PCOLCE2* and *FBLN2*) and cytokine receptors (*OSMR* and *ILST6*), suggesting that they may actively remodel heart structure during development and disease conditions. Furthermore, Wang et al. (2021) performed dual-omics (scRNA-seq and scATAC-seq) on mouse cardiac fibroblasts and discovered new cardiac fibroblast subpopulations with unique functional states. Unexpectedly, Skelly et al. (2018) reported that many other NCMs in the mouse heart expressed some fibroblast marker genes, and they developed a strategy to distinguish real fibroblasts from these stromal cells.

### Spatiotemporal characteristics of fibroblast during embryo heart development

The transcriptome of fibroblasts changes considerably during human heart development and maturation. Cui et al. (2019) constructed the development trajectory of embryonic fibroblasts. They showed that embryonic fibroblasts increased the expression of canonical ECM genes, striated muscle structure development, and differentiation genes but decreased the expression of cell-cycle genes from 5 to 25 PCWs in human hearts. Consistent with that study, Suryawanshi et al. (2020) profiled fetal human heart samples from 19 to 22 PCWs and found that cardiac fibroblasts could be separated into two clusters depending on their proliferation state. The non-proliferating sub-population has higher expression of the ECM gene *DCN*. Similarly, in the first 8 weeks of postnatal murine hearts, fibroblasts were reported to switch from neonatal to the adult state, and gene expression profile reflected enhanced functions related to ECM organization, muscle organ morphogenesis, and BMP signaling pathway (Wang L et al., 2020). Meanwhile, the FABP4^+^ fibroblasts were gradually replaced by the EGR1^+^ subtype, which may contribute to CM maturation (Wang L et al., 2020). Moreover, current findings suggest that a similar transition of fibroblasts may also occur in humans after birth.

In addition to dynamic changes in the transcriptional program during development, fibroblasts also have high heterogeneity depending on their anatomical positions. Spatial transcriptome studies of the human heart revealed that fibroblasts could adopt distinct transcriptional patterns depending on their anatomical position. Asp et al. (2019) systematically conducted scRNA, spatial transcriptomics, and *in situ* sequencing on the developing human heart from 4.5 to 9 PCWs. In their study, embryonic fibroblasts showed more spatiotemporal differences than embryonic cardiomyocytes. The differences between fibroblast subtypes are closely associated with their local microenvironment. For example, fibroblasts localized at the sub-epicardium are involved in connective tissue development and vasculogenesis (Figure 3). Likewise, Cui et al. (2019) found that fibroblasts from distinct chambers have many differentially expressed genes (DEGs).

Consistent with the findings in the fetal heart, adult human cardiac fibroblasts can be separated into region-specific sub-populations based on several marker genes, such as *C7* and *ABCA10* for ventricular fibroblasts, and *GSN* and *FBLN1* for atrial fibroblasts (Tucker et al., 2020). Furthermore, Litviňuková et al. (2020) reported that cardiac fibroblasts could also produce chamber-specific ECMs (*FBLN1* in atria versus *FN1* and *FBN1* in the ventricle). Although most fibroblasts come from the epicardium, lineage tracing studies of the mouse heart showed that the endocardium and neural crest contribute to cardiac fibroblasts at specific anatomical locations (Ivey and Tallquist 2016; Meilhac and Buckingham 2018). Thus, it is highly likely that the developmental origin and local microenvironment caused the transcriptome difference of cardiac fibroblasts at distinct anatomical locations.

The above observations suggest that fibroblasts in the embryonic heart have significant plasticity early on. As the heart morphogenesis proceeds, they gradually exit the proliferation phase and gain a mature state gene expression pattern, marked by higher levels of ECM gene expression. Meanwhile, fibroblasts acquire region-specific transcriptional signatures and express chamber-specific ECM proteins at distinct anatomical positions.

### Transcriptomic change of fibroblast in heart diseases

After injuries, the heart repair process starts immediately. Due to the limited regenerative ability of cardiomyocytes, cardiac interstitial cells are the leading players in injury response (Forte et al., 2020). During both human and mouse heart ischemic injury, cardiac fibroblasts became activated (marked by *POSTN*) and transformed into myofibroblasts (*FAP*, *MYH11*) or fibrocytes (*CHAD*, *COMP*) (Tallquist and Molkentin 2017; Fu et al., 2018; Tucker et al., 2020). Meanwhile, they initiate to produce ECMs and form an adaptive ECM-rich scar to preserve the heart geometry and prevent rupture (Farbehi et al., 2019).

To discover new therapeutic targets for heart diseases, Gladka et al. (2018) performed scRNA-seq on both healthy and ischemic injured adult mouse hearts. They identified cytoskeleton-associated protein 4 (*Ckap4*) as a novel marker for activated fibroblasts. Moreover, the inhibition of *Ckap4* in activated fibroblasts triggered increased expressions of fibrosis-related genes, suggesting a modulating role of *Ckap4* in the activation of fibroblasts. Contradictory to their findings, Farbehi et al. (2019) found that *Ckap4* was also expressed in other cardiac stromal populations based on a single-cell study at multiple time points. They also reported a novel activated fibroblast subtype with a putative role in promoting heart repair, expressing a strong anti-WNT transcriptome signature (*Wif1* positive) in both sham and myocardial infarcted mouse hearts. To elucidate the cellular processes underlying scar formation Forte et al. (2020) perform scRNA-seq of interstitial cells from infarcted mouse hearts carrying a genetic tracer that labels epicardial-derived cells. They characterized fibroblasts from epicardial and endocardial origins and depicted their evolution during the repair process. Interestingly, endocardial-derived cells showed a relatively stable state throughout the repair.

In addition to scRNA-seq, Wang Z et al. (2020) adopted scATAC seq in elucidating the responses of the different cellular components of the mouse heart following injury (Cui, et al., 2020). By comparing the injury responses of non-regenerative and regenerative hearts, they decoded secreted factors, cellular crosstalk, and gene regulatory networks involved in the regeneration process. In particular, they analyzed fibroblast populations’ heterogeneity in shams and injured neonatal hearts (Wang Z et al., 2020; Cui, et al., 2020). Many efforts were made to reveal the mechanisms underlying fibroblast activation. Alexanian et al. (2021) identified MEOX1 as a core transcriptional regulator of fibroblast activation in mouse heart disease (Ruiz-Villalba et al., 2020). Hesse et al. (2021) discovered that some epicardial stromal cells expressed cardiac specification markers and sarcomeric proteins, suggesting they may possess cardiomyogenic potential.

Recently, Tucker et al. (2020) found a subset of fibroblasts with inherently high fibrosis-associated, profibrotic and pathologic remodeling gene expression. These fibroblasts evenly showed up throughout the healthy human heart. This observation indicated a specific cardiac fibroblast population with the potential tendency to turn to an activated state and react to external stimulation in diseases. Through snRNA seq, the unique transcriptional patterns of fibroblasts in distinct human heart diseases were discovered (Chaffin et al., 2022; Hill et al., 2022). Cardiac fibroblasts in hypoplastic left heart syndrome had enrichment for a low HIPPO and high YAP cell state characteristic of activated cardiac fibroblasts but not in Tetralogy of Fallot (Hill et al., 2022). Similarly, activated fibroblasts have different transcriptome signatures in dilated and hypertrophic cardiomyopathy (Chaffin et al., 2022).

In summary, the current single-cell analysis has comprehensively revealed the plasticity of fibroblasts in fetal hearts and their spatiotemporal heterogeneity from embryonic to adult stages. Meanwhile, the dynamic profiles of the cardiac fibroblasts becoming mature or activating in hemostasis or diseased conditions will facilitate researchers acquiring a deeper understanding of the mechanisms underlying heart development and injury repair processes. Although single-cell analyses have revealed the regulators of fibroblast activation and the heterogeneity and flux of cardiac fibroblasts in distinct diseases, further experiments are still needed to identify and confirm their pathological functions.

## Endothelial cells in the heart

As the primary components in the trabecular formation and vessel structure and valves, ECs (*PECAM1*, *CDH5,* and *VWF*) play a fundamental role in heart development and cardiac function (Brade et al., 2013; Günthel, Barnett, and Christoffels 2018). They account for 10%–20% of total cells and decrease during human heart development (Zhou and Pu 2016; Cui et al., 2019). Notably, compared to humans, the proportion of cardiac ECs is higher, and they were reported as the biggest NCM population AuthorAnonymous, 2020b in several studies (Pinto et al., 2016; Li et al., 2016; Wang et al., 2021).

### The heterogeneity of cardiac endothelial cells

**FIGURE 4:**
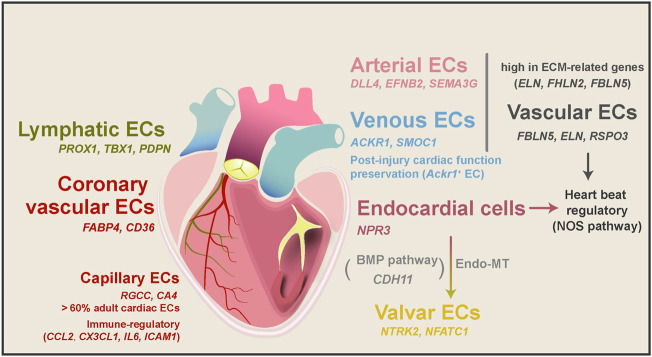
EC subtypes, their transitions, and functions in heart development and diseases. ECs mainly consist of vascular ECs, lymphatic ECs, coronary vascular ECs, capillary ECs, endocardial ECs, and valvar ECs. These subtypes of ECs have different molecular characteristics and biological functions depending on their anatomical positions. Vascular ECs can be divided into arterial and venous ECs, and *Ackr1+* venous ECs have been shown to prepare cardiac function after injury. Both vascular and endocardial ECs were speculated to regulate heartbeat through the NOS pathway. During heart development, parts of endocardial cells transform into valvar cells through the endo-MT process. Capillary ECs are also potential regulators of immune responses in the healthy adult human heart.

Cardiac ECs mainly consist of cells that line the blood vessels, the endocardium, and lymphatic vessels. To explore the heterogeneity of cardiac ECs, Cui et al. (2019) sorted *CD45* ^−^ *CD31* ^+^ cells from the human fetal heart and combined them with other cardiac cells in the following single-cell analyses. From the EC-enriched single-cell transcriptome profile, researchers found that, in 5–7 PCWs human heart, embryonic ECs could be classified into four main subtypes: endocardial ECs, coronary vascular ECs, vascular ECs, and valvar ECs (Figure 4). With a closer spatial examination of these EC subtypes, a spatial atlas of cardiac ECs was drawn (Asp et al., 2019). Endocardial ECs within the endocardium specifically express *NPR3* (Tang et al., 2018; Suryawanshi et al., 2020), while coronary vascular ECs have a specific expression of *FABP4* and *CD36* (Kim and Dyck 2016). In fine detail, ECs in the capillary endothelium of the trabecular myocardium express *TMEM100* uniquely, while *CLDN5* was only found in ECs of endothelium/pericytes/adventia in the compact myocardium (Asp et al., 2019). Depending on their anatomical locations, capillaries supply the blood and oxygen for the trabecular myocardium, while the compact myocardium relies on coronary arteries (Asp et al., 2019). Vascular ECs from the aorta and pulmonary artery vessels have high expression of ECM genes (*ELN*, *FBLN5*, and *FBLN2*) (Scholz et al., 2016; Cui et al., 2019; Suryawanshi et al., 2020), while valvar ECs only appeared in 17 PCWs and older human hearts and marked by high expression of *NTRK2* and *NFATC1* (Wu, Baldwin, and Zhou 2013; Cui et al., 2019).

In the adult human heart, beating CMs have immense oxygen and energy consumption, at which time about 60% of all ECs are capillary ECs (*RGCC* and *CA4*) (Kalucka et al., 2020; Litviňuková et al., 2020). Moreover, vascular ECs can be divided into arterial and venous ECs in the adult human heart. Arterial ECs expressed artery markers *DLL4*, *EFNB2*, and *SEMA3G* (Kashiwazaki et al., 2003; Corada, Morini, and Dejana 2014), while venous ECs expressed vein markers *ACKR1* and *SMOC1* (Kashiwazaki et al., 2003). Interestingly, the mouse heart’s arterial and venous ECs have different DEG patterns (Wang et al., 2021). In addition to these major subtypes, a tiny proportion (∼1%) of the adult human cardiac ECs are lymphatic ECs marked by *PROX1*, *TBX1*, and *PDPN* (Kalucka et al., 2020; Tucker et al., 2020), similar to their RNA Epelman et al., 2014b signatures in the mouse hearts (Pinto et al., 2016; Wang et al., 2021). Lymphatic ECs may develop from a migratory EC subtype in the human fetal heart, as they highly express lymphocyte migration genes (*CD44* and *SELL*) (Singh, Erb, and Zöller 2013; Suryawanshi et al., 2020).

Several studies also reported the spatiotemporal transcriptional changes of ECs from the embryonic to adult stage in mouse hearts (DeLaughter et al., 2016; Li et al., 2016; Skelly et al., 2018; de Soysa et al., 2019). Moreover, epigenetic profiling and pseudo-time analysis were used in reconstructing the developmental trajectories of ECs from cardiac progenitor cells and characterizing ECs’ molecular and cellular features in the mouse heart (Jia et al., 2018; Lescroart et al., 2018; Wang et al., 2021). These epigenetic studies provided a new layer of information about endothelial cell fate specification. For example, integrative analysis of scRNA-seq and scATAC-seq suggested that the induction of the Sox gene family may promote cardiac progenitor cells to take endothelial fate (Jia et al., 2018).

As specialized ECs, endocardial cells transform into heart valves during heart development through endothelial-mesenchymal transformation (endo-MT) (Zhang, Lui, and Zhou 2018). The heart valves play a crucial role in blood circulation and prevent blood from flowing back. They are composed of valvar ECs (*CDH5*, *TIE1*, and *TEK*) and valvar interstitial cells (*CDH5* ^*−*^, *PECAM1* ^*−*^, *TIE1* ^*−*^) (Hinton et al., 2006; Cui et al., 2019). In addition to dramatic ECM remodeling, Cui et al. (2019) found apoptosis-related genes were upregulated in parts of valvar cells from 20 PCWs to around 25 PCWs, consistent with the presence of Caspase three protein. This observation suggests that valve development may involve endo-MT and cell apoptosis. Moreover, ligand and receptor analysis also identified BMP signaling pathway as a potential regulator of human endo-MT (Cui et al., 2019).

Single-cell analyses revealed interesting differences between mouse and human cardiac ECs. For example, *CDH11,* a regulator of endo-MT and valve formation, is restricted to the valves of mice and chicks but enriched in human endocardial cells (Bowen et al., 2015; Cui et al., 2019). Likewise, *NTRK2,* which regulates blood vessel formation, only exists in mouse vascular EC (Wagner et al., 2005), and was highly expressed in human embryonic heart valves (Cui et al., 2019). The above two observations hint at the species differences in cardiac valve development.

### Endothelial cells in heart homeostasis maintenance and disease conditions

ECs are critical regulators of cardiac homeostasis maintenance Epelman et al., 2014 and developmental processes. Suryawanshi et al. (2020) discovered the activation of the NOS pathway genes in parts of human cardiac endocardial and vascular ECs. The NOS pathway regulates many essential cardiovascular functions in cardiovascular development, including Ca^2+^ influxes and homeostasis, sarcomere Ca^2+^ sensitivity, and mitochondrial respiration (Farah, Michel, and Balligand 2018). Thus, cardiac ECs in distinct structures may have different functional states. Immune regulation and antigen presentation-related genes (*CCL2*, *CX3CL1*, *IL6*, and *ICAM1*) were high in capillary ECs in the adult human heart (Raemer et al., 2009; Pober et al., 2017), which indicates ECs may involve in immune regulation of the heart (Litviňuková et al., 2020). Furthermore, embryonic cardiac ECs were reported to regulate the compaction of the myocardium by the NOTCH signaling pathway (Cui et al., 2019).

The single-cell analyses have also been used to discover the candidate genes in ECs implicated in adult heart disease and repair. Wang L et al. (2020) compared the cell atlas of the normal mouse heart and heart failure caused by dilated cardiomyopathy or coronary heart disease. They found a significant decrease in the ACKR1^+^ EC subtype in coronary heart disease (Yu et al., 2020). Moreover, injecting ACKR1^+^ ECs into myocardial infarcted hearts preserved cardiac function. Chaffin et al. (2022) also reported the compositional change of ECs in dilated and hypertrophic cardiomyopathy using snRNA-seq. Interestingly, they observed an increase in angiogenic-like EC population in the above heart diseases. The single-cell transcriptional and epigenetic analyses have been used to study the change in ECs during the mouse heart injury-repair process (Farbehi et al., 2019) and neonatal mouse heart regeneration (Wang Z et al., 2020; Cui, et al., 2020). Tang et al. (2022) analyzed the cell type and state changes of graft infiltrating cells in acute mouse heart transplant rejection. They found a specific EC population (positive for *Vcam1*and *Ubd*) which may mediate the rejection by activating the immune system. Hill et al. (2022) profiled ECs in congenital heart diseases (CHDs) through snRNA seq. Unexpectedly, ECs appeared more homogenous across CHDs (including hypoplastic left heart syndrome and Tetralogy of Fallot) compared to differences in CMs and fibroblasts. However, the implication of this observation is unclear.

In summary, spatiotemporal single-cell analysis of cardiac ECs revealed the position-specific and especially function-specific transcriptional signatures of ECs in distinct developmental stages. Their involvement in regulating heart development, homeostasis maintenance, and injury repair was discovered through cellular component analysis, trajectory reconstruction, and ligand and receptor analysis. This knowledge provides valuable insight into understanding the myocardium compaction and valve formation of the heart during embryo development and new clues to preserve CM functions and modulate immune systems in disease conditions.

## Macrophages in the heart

Single-cell RNA-seq revealed that 5%–20% of cells in the human heart are immune cells depending on the stage of development, anatomical location, and the method to harvest the cell (Cui et al., 2019; Litviňuková et al., 2020; Suryawanshi et al., 2020). Cardiac immune cells include macrophages, monocytes, B cells, T cells, and mast cells. Single-cell transcription factor (TF) enrichment analysis revealed that macrophages, monocytes, B cells, T cells, and mast cells highly express immune-cell-related TFs *MAF*, *BATF3*, *BCL11A*, *GATA3*, and *GATA2*, respectively (Suryawanshi et al., 2020). Single-cell study of 19–22 weeks human fetal heart by Suryawanshi found that among heart immune cells (22.7% of total cells), the largest populations were macrophages (4.7% of total cells) and monocytes (4.3% of total cells) (Suryawanshi et al., 2020). Macrophages have been shown to play a central role in heart immune responses to injury, evidenced by their immune modulation, fibrosis regulation, phagocytosis, and angiogenesis-promoting abilities (Pinto, Godwin, and Rosenthal 2014). Single-cell studies of precious human samples highlighted the heterogeneity of macrophages in the heart. On the other hand, lineage tracing and heart injury-recovery experiments using the mouse model revealed the source of cardiac macrophage heterogeneity and the dynamic change in their population and transcriptome during different phases of diseases.

### The origins of macrophages in the mouse heart and their heterogeneity in the human heart

**FIGURE 5:**
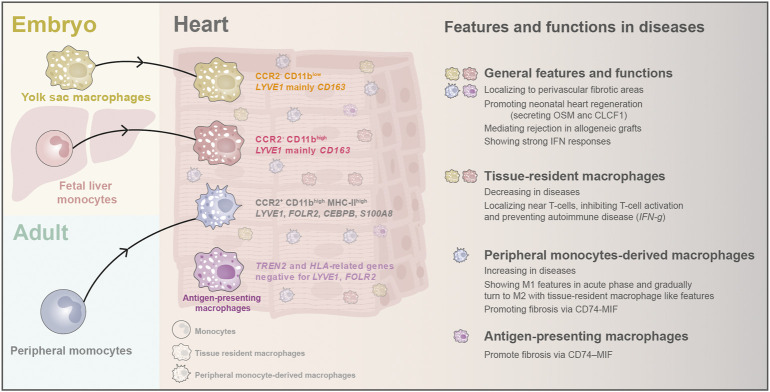
The origins and classification of macrophages and their newly discovered features and biological functions in heart diseases. In early embryos, yolk sac macrophages and fetal liver monocytes arrive the hearts and stay as tissue-resident macrophages. After birth, monocytes from peripheral blood emigrate into the heart and become another critical part of cardiac macrophages. Recently, scRNA-seq found a novel antigen-presenting macrophage population in adult human hearts. Cardiac macrophages have been found to localize to perivascular fibrotic areas after injury, and they can promote CM regeneration in neonatal hearts. They are also activated in the graft rejection process and can induce strong IFN responses. Macrophages display different features and functions in heart diseases depending on their origin.

Cardiac macrophages express typical surface markers CD14, CD68, and CSF1R. They consist of three populations with discrete ontological origins, including primitive yolk sac-derived macrophages (CD11b^low^), fetal monocyte-derived macrophages (CD11b^high^), and adult monocyte-derived macrophages (CD11b^high^) (Yona et al., 2013; Bajpai et al., 2018; Lavine et al., 2018). Lineage tracing study in mice found that the yolk sac and fetal monocyte-derived macrophages arrive at the heart through circulation at E11.5 and E14.5 and are maintained independently of peripheral monocyte input through self-proliferation in adulthood (Hashimoto et al., 2013; Epelman et al., 2014; Lavine et al., 2018). After birth, the proportion of embryo-derived cardiac residential macrophage gradually declines. Meanwhile, the peripheral blood monocyte-derived macrophages start to penetrate the hearts and make up the third cardiac macrophage population (Hashimoto et al., 2013; Yona et al., 2013; Epelman et al., 2014; Lavine et al., 2014) (Figure 5). In the adult mouse heart, cardiac macrophages can be classified into two subsets: CCR2^-^ and CCR2^+^. The CCR2^-^ subset is defined as tissue-resident macrophages derived from embryo origins. In contrast, the CCR2^+^ MHC-Ⅱ^high^ subset is primarily derived from adult peripheral monocytes and acquires subsequent input through a CCR2-dependent mechanism (Lavine et al., 2014; Lavine et al., 2018). This classification method has been supported by the scRNA-seq study in the adult mouse (Hulsmans et al., 2017). The CCR2^-^ and CCR2^+^ populations have distinct functions in the heart. The resident CCR2^-^ macrophages regulate heart repair and coronary development with enhanced phagocytosis ability, while CCR2^+^ macrophages drive inflammation in the heart and cause cardiac dysfunction (Lavine et al., 2014; Leid et al., 2016). Furthermore, cumulative evidence has demonstrated that adult monocyte-derived macrophages mainly induce inflammation responses after injury, while tissue-resident macrophages express fewer inflammatory mediators and have less capacity for secreting inflammatory chemokine and cytokine (Epelman et al., 2014; Epelman, Lavine, and Randolph 2014; Lavine et al., 2014).

In the human hearts, the single-cell study has provided new knowledge about the diversity of cardiac macrophages and their unique characteristics: tissue-resident Macrophages are positive for *LYVE1 and CD163* (Lim et al., 2018; Tucker et al., 2020); peripheral monocyte-derived macrophages express monocyte-like markers (*LYVE1*, *FOLR2*, *CEBPB*, and S100A8) (Nielsen, Andersen, and Møller 2020); while antigen-presenting macrophages were enriched for *TREN2* and *HLA*-related genes but negative for *LYVE1*, *FOLR2*, and *MERTK* (Jaitin et al., 2019; Litviňuková et al., 2020). Moreover, each population can be split into several tiny aggregates by sub-clustering analysis, but these aggregates lack specific functional annotations currently (Litviňuková et al., 2020; Tucker et al., 2020). Curiously, the transcriptome of macrophages in the right atrial differed from that in other chambers, and the reason remains to be investigated (Tucker et al., 2020).

### The roles of macrophages in heart homeostasis and diseases revealed by single-cell studies

Traditional biological studies have revealed that cardiac macrophages have a wide range of functions in heart development and diseases, such as: regulating coronary development and remodeling by secreting IGF-1/2 and VEGF (Wu et al., 2010; Leid et al., 2016); governing myocardial neutrophil infiltration after ischemia-reperfusion injury (Li, Hsiao, et al., 2016); facilitating electrical conduction by forming gap junctions with CMs (Hulsmans et al., 2017). Empowered by single-cell analysis technology, many novel biological and pathological functions of macrophages have been further discovered.

During homeostasis, LYVE1^+^, monocyte-derived macrophages are predicated on interacting with fibroblasts *via* CD74–MIF, and inhibiting this interaction can lead to fibrosis (Litviňuková et al., 2020). Furthermore, macrophages’ cellular and molecular transition and responses in different human congenital and adult heart diseases have been comprehensively depicted (Suryawanshi et al., 2020; Wang L et al., 2020; Yu, et al., 2020; Chaffin et al., 2022; Hill et al., 2022). In the congenital heart block, Suryawanshi et al. (2020) found that human cardiac macrophages in disease showed greater interferon (IFN) responses than cells isolated from the health. Through snRNA-seq and imaging mass cytometry, Hill et al. (2022) found cardiac macrophages localized to perivascular fibrotic areas in the CHDs. They also found an immuno-suppressive macrophage population localized near T-cells with IFN-gamma signaling pathway genes and PD-L1 expression. These PD-L1 high macrophages might inhibit T-cell activation and prevent autoimmune responses in the heart. Interestingly, a reduction of proliferative macrophages was observed in both CHDs and adult heart diseases (Chaffin et al., 2022; Hill et al., 2022). This observation supports the notion that in adult hearts, during disease circumstances, the replenishment of macrophages relies on monocyte recruitment rather than local proliferation. The proliferative macrophages in adult heart diseases have been demonstrated as the CCR2^-^ populations (Chaffin et al., 2022), and we believe it is the same in CHDs.

Similar observations were reported in the mouse single-cell studies with more detail (Dick et al., 2019; Farbehi et al., 2019). Dick et al. (2019) found that Ischemic injury reduced resident macrophage abundance, whereas CCR2^+^ monocyte-derived macrophages expanded and adopted multiple cell fates within infarcted tissue, including those nearly indistinguishable from resident macrophages . Likewise, in the study of Farbehi et al. (2019) the more abundant population at MI-day 3 was identified as classical monocyte-derived M1 macrophages (Ccr2^high^ MHC-II^+^Ly6c2^+^). In contrast, the most prominent population at MI-day 7 was identified as non-classical M2 macrophages (Ccr2^high^ MHC-II^+^Ly6c2^-^) involved in inflammation resolution and repair. Farbehi et al. (2019) also mapped a continuum of state transition trajectory from monocytes through M1 macrophages to M2 macrophages across the injury response, consistent with the well-recognized plasticity of peripheral monocytes. Their trajectory analysis also showed the convergence of M2 macrophages with tissue-resident macrophages. Moreover, another subsequent study by Ren et al. (2020) revealed that activating proinflammatory macrophages in cardiac hypertrophy is crucial in the transition from normal to reduced cardiac function, and inhibiting inflammation during the later stage of cardiac hypertrophy can help slow down the progression of hypertrophy. Although all the above studies emphasized the importance of peripheral monocyte-derived macrophages in the injury-repair process, the presence of resident macrophages Li et al., 2016b is also crucial, as their depletion can lead to impaired cardiac function (Dick et al., 2019).

Through scRNA-seq and scATAC-seq, Wang Z et al. (2020) found that during murine heart regeneration, macrophages upregulated *Clcf1* expression. To investigate the potential role of macrophages in promoting heart regeneration, they overexpressed Clcf1 using recombinant adenovirus and found a significantly enhanced proliferation of CMs *in vitro*. Together, their work suggested that the macrophage-enriched secreted factor CLCF1 can promote CM proliferation during neonatal heart regeneration (Wang Z. et al., 2020). Moreover, researchers found that the immune activation in the macrophages can lead to the upregulation of oncostatin M (*OSM*) in proinflammatory Li et al., 2016 macrophages upon induction of pressure overload (Martini et al., 2019). Excitingly, Li et al. (2020) found that macrophages can promote neonatal mouse heart regeneration through secreting OSM with the activation of gp130 on the CM surface. As well-known, adult hearts have little regenerative potential. Thus, the finding of both OSM and CLCF1 have provided valuable potential therapeutic targets to improve heart regeneration after cardiac injury.

Rejection response is an important research topic in heart transplantation. Recently, researchers used scRNA-seq to analyze cell composition and state changes after heart transplantation (Tang et al., 2022). By comparing the immune cell population between allogeneic and syngeneic samples, Tang et al. (2022) found that infiltrating macrophages were predominantly from allogeneic grafts. Furthermore, only one infiltrating macrophage cluster was in an active state with the upregulation of *CD40*, *Fam26f,* and *Pira2*, suggesting they may be the specific population mediating rejection.

Collectively, the analysis of macrophages from single-cell studies has provided much information about their developmental origin and potential function during heart formation and pathological situations. This knowledge will help find new therapeutic venues to control the inflammation response better and promote heart repair.

## Non-cardiomyocytes subtypes account for less than 5% of the total cell population

### Smooth muscle cells

Smooth muscle cells (SMCs) (*ACTA2*, *RGS5*, and *TAGLN*) are an essential component of the cardiac vessel walls, supplying the dominant structure support of the cardiac outflow tract (OFT). Asp et al. (2019) deconvolved the cellular heterogeneity in bulk *in situ* RNA sequencing of the human embryonic heart and found spatial-specific gene patterns of SMCs and mesenchymal cells at different locations (Figure 3). Furthermore, their spatial transcriptome analysis revealed that SMC marker genes start to be expressed in the distal part of the atrioventricular mesenchyme of the human heart from 4.5 to 5 PCWs (Asp et al., 2019).

Human SMCs show an immature state at embryonic stages compared to adult stages, evidenced by their specific expression of *PDGFRL*, *SFRP2,* and *CPZ* and limited expression Wang L et al., 2020 of *MYH11*, *ACTA2,* and *TAGLN* (Owens, Kumar, and Wamhoff 2004; Cui et al., 2019). Compared to fibroblasts, SMCs express higher levels of insulin-like growth factor (IGF-1) in the embryonic human heart (Suryawanshi et al., 2020), suggesting that they may promote the growth of the embryonic heart and great vessels since IGF-1 is a crucial factor for CM proliferation (Xin et al., 2011). In the adult human heart Wang Z et al., 2020, two SMC subtypes could be distinguished: one expresses relatively high levels of *ACTA2*, *CNN1*, and *TAGLN*, indicating an arterial origin; the other expresses *LGR6*, a stem-cell marker gene (Barker and Clevers 2010), suggesting their relatively immature status and their stem cell-like property or plasticity (Litviňuková et al., 2020). The state of SMCs is also affected by diseases. In coronary artery atherosclerosis, SMCs can transform into intermediate cell types with transcriptomes similar to stem cells, endothelial cells, or monocytes under the control of retinoic acid signaling (Pan et al., 2020).

### Pericytes

Pericytes (*ABCC9* and *KCNJ8*) are considered mural cells together with SMCs, and they closely interact with ECs to regulate vasculature development (Nelson et al., 2015; Hosford et al., 2019; Kirkwood et al., 2021). Pericytes in the atria and ventricle have different transcriptional signatures: *ACTA2* and *TAGLN* are highly expressed by atria pericytes, while ventricular pericytes have high levels of *RGS5*, *ABCC9*, and *NCAM2* (Asp et al., 2019). Moreover, Litviňuková et al. (2020) defined a new sub-cluster of pericytes in a transition state from or towards EC by RNA velocity analysis. This new cluster expressed both pericyte and pan-EC markers. This finding indirectly supported the theory that ECs might be progenitors of cardiac pericytes and provided further evidence for bidirectional trans-differentiation between ECs and pericytes (Chen et al., 2016).

### Epicardial cells

As a possible *in vivo* cell source to replenish lost cardiomyocytes, *Wt1* ^*+*^ EPCs (*ALDH1A2*, *LRP2,* and *ITLN1*) have been shown to differentiate into cardiomyocytes during mouse heart development and are proposed to have cardiomyogenic potential in the adult heart (Cai et al., 2008; Zhou et al., 2008; González-Rosa, Peralta, and Mercader 2012; Suffee et al., 2020). In the embryonic human heart, the number of EPCs increased from 0.5% (5–7 PCWs) to 5% (25 PCWs) of total cells (Cui et al., 2019). Like other NCM cell types, epicardial cells also have spatial-specific characteristics. Spatial transcriptome and single-molecular fluorescence *in situ* hybridization studies revealed that epicardial cells located at the epicardial layer are enriched for *ITLN1* while the atrioventricular sub-epicardial mesenchyme has higher *TBX18* expression (Asp et al., 2019; Litviňuková et al., 2020).

### Neuronal cells

The heart conduction system governs heart beating rhythm, while neuronal cells, the core of the conduction system, only account for no more than 1% of cells in the human heart (Litviňuková et al., 2020). Neuronal cells in the embryonic human heart include Schwann cells and cardiac neural crest cells. Cardiac neural crest cells (*ISL1* and *STMN2*) emerge at the mediastinal mesenchyme and OFT in 4.5-5 PCWs, followed by the appearance of Schwann progenitor cells (*ALDH1A1*) at the mediastinal mesenchyme, OFT, and AV sub-epicardial mesenchyme in 6.5 PCWs human hearts (Brade et al., 2013; Asp et al., 2019). Interestingly, in the adult human heart, the single-cell analysis found that some neuronal cells express both the central nervous system marker (*PRKG1*) and EC genes (Struk et al., 2019; Litviňuková et al., 2020). However, as the number of such neuronal cells is meager, this observation needs further validation to rule out errors caused by techniques such as doublet. Moreover, the biological significance behind it remains to be discovered.

## Conclusion

Single-cell analyses have provided a significant amount of new knowledge about the NCMs during cardiac development and in diseased hearts. These NCMs come from different origins and acquire regional and function-specific transcriptome signatures during development. Moreover, their transcriptome changes significantly in various disease conditions. The same NCMs types in mice and humans shared similar marker genes, although some species-specific gene expression can be detected (Cui et al., 2019). Human NCMs also displayed conserved change patterns associated with gender and age (Litviňuková et al., 2020). An increasing number of studies use mouse models and human samples plus single-cell analysis to discover new mechanisms to regenerate the heart. ScRNA-seq analyses also found that the intermediate state exists in many NCM types, suggesting the highly dynamic nature of these cells in the heart. Nevertheless, owing to the rare population of such cells and intrinsic technical deficiencies of scRNA-seq (e.g., doublet), further experiments are required to validate these findings, such as dual-lineage tracing and systematic quantification of dual-cell type marker genes.

Recent advances in spatial transcriptomics and single-cell multi-omics, including epigenomics, and proteomics, added even more layers of information. Integrative analysis of the single-cell data from different studies and biological contexts will provide many venues to discover new cell subtypes, novel biomarkers, and therapeutic targets for treating cardiovascular diseases.
